# Cardioprotective effects of puerarin against myocardial ischemia–reperfusion injury: a preclinical systematic review and meta-analysis

**DOI:** 10.3389/fphar.2026.1770407

**Published:** 2026-03-17

**Authors:** Ding Chen, Cong Xue, Zheng Liang, Xingye Wang, Zhao Shi, Ming Yao, Yiqiang Wang, Lihong Jiang

**Affiliations:** 1 College of Traditional Chinese Medicine, Changchun University of Traditional Chinese Medicine, Changchun, Jilin, China; 2 Heart Disease Center, Hospital Affiliated to Changchun University of Traditional Chinese Medicine, Changchun, Jilin, China

**Keywords:** animal model, meta-analyses, myocardial ischemia–reperfusion, puerarin, systematic review

## Abstract

**Background:**

Myocardial ischemia–reperfusion injury (MIRI) remains a pivotal clinical conundrum in clinical cardiovascular practice, accounting for a substantial proportion of morbidity and mortality associated with cardiovascular disorders. Puerarin, a natural isoflavone derived from kudzu root, has shown promising cardioprotective potential in preclinical studies.

**Methods:**

Relevant studies were systematically searched in PubMed, Embase, Cochrane Library, China National Knowledge Infrastructure (CNKI), Wanfang, VIP Database, and Web of Science from inception to October 2025 for preclinical studies evaluating puerarin’s effects on MIRI. Key outcome measures included myocardial infarction size, myocardial ischemic size, cardiac function parameters, myocardial injury markers, oxidative stress indicators, inflammatory cytokines, and the cardiomyocyte apoptosis index. Methodological quality was assessed using the SYRCLE risk-of-bias tool and GRADE tool, and meta-analyses were performed with RevMan 5.4.1 and STATA 18.0.

**Results:**

A total of 29 eligible studies were included. This meta-analysis showed that puerarin administration reduced the myocardial infarction size and myocardial ischemic size, improved cardiac systolic/diastolic function (e.g., increased LVEF, LVSP, and LVFS; decreased LVIDd and LVEDP), attenuated myocardial injury (decreased cTn-T, CK, CK-MB, and LDH levels), suppressed oxidative stress (elevated SOD and NO; reduced MDA), inhibited inflammatory responses (decreased TNF-α, IL-1β, and IL-6; increased GSH), and reduced cardiomyocyte apoptosis. Subgroup analysis indicated potential influences of the administration route, dosage, and animal body weight on partial outcomes.

**Conclusion:**

Preclinical evidence demonstrates that puerarin exerts cardioprotective effects against MIRI through multi-target mechanisms, including mitigating oxidative stress, suppressing inflammation, and inhibiting cardiomyocyte apoptosis. Despite consistent preclinical efficacy, well-designed clinical trials are needed to validate its translational potential and safety in humans.

**Systematic Review Registration:**

https://www.crd.york.ac.uk/PROSPERO/view/CRD420251168227, identifier CRD420251168227.

## 1 Introduction

Myocardial ischemia–reperfusion injury (MIRI) refers to the phenomenon wherein myocardial tissue, upon the restoration of blood flow after a period of ischemia, exhibits exacerbated ischemic damage. MIRI can lead to abnormalities in myocardial systolic or diastolic function, abnormal electrical discharges around necrotic foci, and malignant arrhythmias, significantly impairing cardiac function. The in-hospital mortality rate for patients suffering from myocardial infarction complicated by MIRI ranges from 6% to 14% (Xiang et al., 2024; Keeley et al., 2003). This injury is a pathophysiological process resulting from the interplay of multiple mechanisms, including inflammatory responses, oxidative stress, cardiomyocyte apoptosis, and necrosis; however, its exact pathogenesis has not been fully elucidated (Sánchez-hernández et al., 2020). Acute myocardial infarction (AMI), a leading cause of death in coronary heart disease, is primarily characterized by acute coronary thrombosis due to atherosclerotic plaque rupture or erosion, leading to myocardial necrosis. Reperfusion therapy, including thrombolysis, percutaneous coronary intervention, or surgery, is a critical intervention for AMI, aimed at revascularization and salvaging jeopardized myocardium (Ibanez et al., 2018; Al et al., 2019). However, some patients experience MIRI following blood-flow restoration, which paradoxically aggravates myocardial damage. Therefore, mitigating MIRI and improving patient outcomes have become urgent clinical challenges (Zheng et al., 2025). Current pharmacological strategies for MIRI mainly include antioxidants, calcium channel blockers, Na+/H+ exchange inhibitors, and angiotensin-converting enzyme inhibitors. Most of these agents target single pathways involved in MIRI, offering limited intervention and often suboptimal clinical efficacy (Qi et al., 2017).

Puerarin, also known as 7,4′-dihydroxy-8-C-β-D-glucopyranosyl isoflavone, is the primary bioactive component extracted from *Pueraria lobata* (Liu et al., 2019), and recent *in vivo* and *in vitro* studies have provided substantial evidence supporting its significant cardioprotective effects against MIRI. Notably, puerarin has been shown to reduce myocardial infarction size, improve coronary blood flow, and enhance myocardial oxygen metabolism (Jia et al., 2024). Its protective mechanisms are multifaceted and primarily include the following: antioxidant effects, achieved by enhancing the activity of antioxidant enzymes such as SOD, CAT, and GSH-Px, as well as activating the Nrf2 pathway to mitigate oxidative stress (Xu et al., 2019; Zhang et al., 2023); anti-inflammatory actions, mediated through the suppression of pro-inflammatory cytokines (IL-6 and TNF-α) and inhibition of the NF-κB and SIRT1 signaling pathways; regulation of autophagy, by modulating autophagy-related proteins (BAG3, Beclin-1, and LC3II) and the Akt pathway to maintain autophagic balance and prevent excessive autophagy-induced cell death (Tang et al., 2017; Ma et al., 2016); and inhibition of ferroptosis, via reducing iron accumulation and lipid peroxidation, thereby preserving mitochondrial integrity and cell viability (Ding et al., 2023; Zhou et al., 2022). These mechanisms collectively underlie its roles in anti-myocardial fibrosis, inhibition of inflammatory infiltration, and protection against endothelial injury, thereby conferring cardiovascular protection (Duan et al., 2000; Pan et al., 2024; Sun and Jin, 2017). Therefore, puerarin also demonstrates a potential for the treatment of MIRI. Puerarin injections have been used clinically for conditions such as coronary heart disease (Shi et al., 2002), retinal artery and vein occlusion (Meng et al., 2022), and sudden hearing loss (Xie et al., 2018), and their long-term efficacy and safety need further validation. Given the complexity of puerarin’s mechanisms in MIRI and the involvement of multiple cellular signaling pathways, future research should continue to elucidate its mechanisms, develop derivatives, and improve techniques to enhance its bioavailability, thereby providing new strategies and evidence for MIRI treatment, and its inherent chemical structure, issues related to administration routes, and bioavailability require further clarification.

**FIGURE 1:**
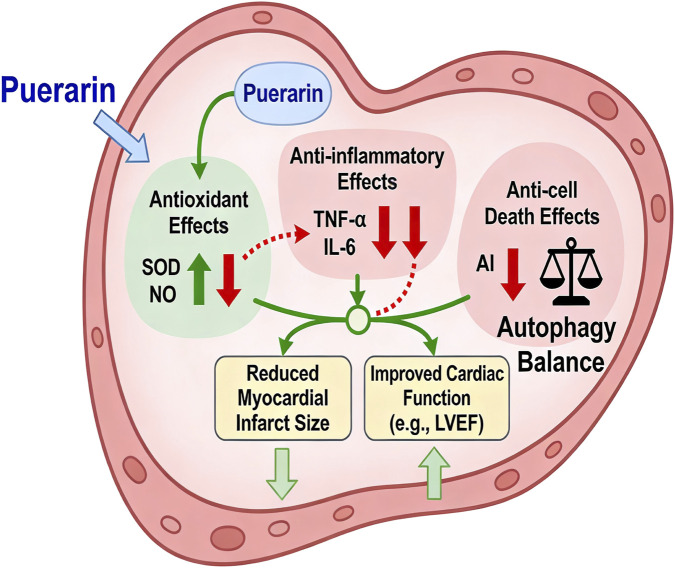
Pharmacological effects of puerarin.

Collectively, preclinical evidence indicates that puerarin holds promise as a multi-target cardioprotective agent against MIRI, exhibiting a range of pharmacological properties—including antioxidant, anti-inflammatory, autophagy-modulating, and anti-ferroptotic activities—through mechanisms that diverge from those of conventional therapies (Figure 1). Although several narrative reviews (Jia et al., 2024) have summarized these potential mechanisms from a pharmacological perspective, a comprehensive quantitative synthesis of the robust preclinical evidence is currently lacking. Narrative reviews are valuable for hypothesis generation but cannot provide quantified effect estimates or systematically explore the sources of heterogeneity across studies. In contrast, systematic reviews and meta-analyses of animal studies offer a higher level of evidence by statistically integrating results from multiple independent experiments, enabling a more robust estimation of overall efficacy, identification of consistent effects across different experimental settings, and exploration of influencing factors through subgroup analysis. However, given that previous studies were conducted some time ago, and considering the continuous publication of numerous relevant experimental studies (Wenjun et al., 2015) since then, in this paper, we incorporate more recent literature along with additional cardiovascular outcome indicators. Therefore, the present review provides a more comprehensive synthesis than previous reports.

## 2 Methods

### 2.1 Literature search

A systematic literature search was conducted across seven electronic databases—PubMed, Embase, the Cochrane Library, China National Knowledge Infrastructure (CNKI), China Science and Technology Journal Database, Wanfang, and Web of Science—to identify animal studies evaluating the effects of puerarin on MIRI. The search encompassed literature from the inception of each database up to October 2025. The retrieval strategy utilized a combination of subject headings and keywords, including “Puerarin” and “Myocardial Reperfusion Injury.” Within each conceptual category, relevant subject terms and free-text words were combined using the Boolean operator “OR,” and the three sets of terms were subsequently intersected using “AND” (Prill et al., 2021). This systematic review and meta-analysis adhered to the Preferred Reporting Items for Systematic Reviews and Meta-Analyses (PRISMA) 2020 guidelines (Page et al., 2021), thereby ensuring methodological rigor, transparency, and reproducibility. The study protocol was prospectively registered on the PROSPERO international prospective register of systematic reviews. The registration number is CRD420251168227 https://www.crd.york.ac.uk/PROSPERO/view/CRD420251168227.

### 2.2 Inclusion and exclusion criteria

#### 2.2.1 Inclusion criteria

Studies were included if they met the following criteria:

1. Animal model: the study must utilize an animal model of myocardial ischemia–reperfusion (I/R) injury. The methodology for inducing I/R (coronary artery ligation) must be well established and appropriately described.
2. Intervention: the intervention in the experimental group must be puerarin or a puerarin-based preparation. Co-administration with other cardioprotective drugs in the experimental group was not permitted. The control group must receive either no treatment or a placebo (saline or vehicle).
3. Outcome measures: the study must report quantitative data on efficacy metrics related to myocardial I/R injury that are aligned with the protocol, while maintaining the original outcome measures and incorporating additional relevant indicators to enhance the robustness of the findings. Key outcome measures (myocardial infarction size, cardiac enzyme levels, hemodynamic parameters, and histopathological scores) must be presented in a form that allows for the direct or indirect extraction or calculation of the means and standard deviations (or standard errors).
4. Publication type: only original research articles published in English or Chinese were considered.

#### 2.2.2 Exclusion criteria

Studies were excluded based on the following criteria:

1. Species: studies using other laboratory animals such as rabbits, zebrafish, and *Drosophila*, among others.
2. Studies with incomplete outcome data, data presented only in the graphical form without accessible numerical values, or data that could not be reliably extracted or calculated.
3. Article type: reviews, meta-analyses, systematic reviews, conference abstracts, editorials, or case reports.
4. Publications in languages other than English or Chinese.
5. Repeatedly published literature.
6. Preclinical studies that were inconsistent in the study purpose.

### 2.3 Data extraction

Following the literature search, duplicates, non-full-text entries, and studies failing to meet the inclusion criteria were excluded. Three independent investigators (DC, MY, and CX) extracted the following data from the eligible publications: first author, publication year, animal species, modeling methodology, sample size, administration route, dosage and timing, numerically measured outcomes, and any extractable results. The average discrepancy between the two reviewers’ extractions was maintained below 1%. Any inconsistencies were resolved through discussion until a consensus was reached. The agreed-upon data were subsequently reviewed by four additional investigators (ZL, X-Y W, Y-Q W, and L-H J). In cases where disagreements persisted, a third reviewer (ZS) performed a final extraction to reach a definitive conclusion. For critical missing data, corresponding authors were contacted *via* email to request the necessary information. Finally, all outcome indicators intended for meta-analyses were systematically summarized.

### 2.4 Quality assessment

To evaluate the methodological rigor of the included studies, two independent reviewers (DC and CX) appraised the risk of bias using the 10-item SYRCLE risk-of-bias tool (Hooijmans et al., 2014). This instrument examines key domains such as selection bias (covering sequence generation, allocation concealment, and random housing), performance bias, detection bias (including random outcome assessment and blinding), attrition bias, reporting bias, and other potential sources of bias. Each criterion was classified as “low risk,” “high risk,” or “unclear risk.” Any disagreements arising during the quality evaluation were resolved through discussion with a third reviewer (ZL) to achieve consensus.

### 2.5 Statistical analysis

Statistical analyses were conducted using Review Manager (RevMan) version 5.4.1 and STATA version 18.0. The heterogeneity of the included studies was assessed using the I^2^ test. Given the anticipated clinical and methodological diversity among animal studies, we utilized the random-effects model for all meta-analyses to provide a more conservative and generalizable estimate of the mean effect. Substantial heterogeneity was defined as I^2^ > 50%. To explore potential sources of high heterogeneity (I^2^ > 75%), we performed subgroup analyses. Sensitivity analysis was conducted by sequentially removing each study to verify the robustness of the results and to explore potential sources of heterogeneity. Statistical significance was defined as P < 0.05. When 10 or more studies reported the same outcome indicators, Egger’s test was used to assess the potential publication bias.

## 3 Results

### 3.1 Study selection

**FIGURE 2:**
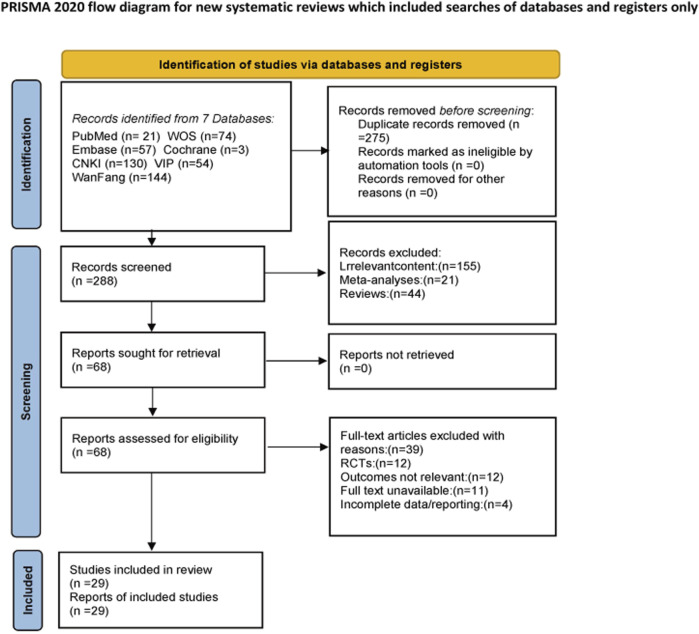
PRISMA flow diagram of study selection and inclusion.

The initial literature search retrieved a total of 563 records from the following databases: CNKI (n = 130), CBM (n = 80), VIP (n = 54), Wanfang (n = 144), PubMed (n = 21), Embase (n = 57), Cochrane Library (n = 3), and Web of Science (n = 74). After removing 275 duplicates, 288 records remained for screening. During the screening phase, 220 records were excluded for the following reasons: irrelevant content (n = 155), meta-analyses (n = 21), and reviews (n = 44). Subsequently, 68 full-text articles were assessed for eligibility. After a detailed evaluation of the full-text articles, 29 studies met the inclusion criteria and were included in the systematic review, and the specific screening process is shown in Figure 2.

### 3.2 Article features

A total of 29 studies were included in the analysis, and they utilized two laboratory animal species: rats (27 studies) and mice (two studies). Three distinct strains were reported: Sprague–Dawley (SD) rats (n = 23), Wistar rats (n = 4), and C57BL/6 mice (n = 2). Group sizes ranged from 3 to 20 animals per experimental or control group. Animal ages varied between 6 and 10 weeks. The majority of studies (n = 25) used only male animals; four studies included both male and female subjects, with only one study using females exclusively. Among the 22 studies that reported body weights, values were predominantly maintained within 200 g–350 g, whereas seven studies did not report weight data. Reported weights varied across studies, which was largely due to differences in species and strain (detailed characteristics are provided in Table 1).

**TABLE 1:** Characteristics of articles.

No.	Study ID	Animals	Modeling method	No. Of animals (control group/Puerarin group)	Administration route	Dosage	Drug administration time	Outcome indexes	Mechanistic pathway(s)
1	Ding et al. (2023)	Male C57BL/6 mice	Ligature LAD 30 min, reperfusion 24 h	6/6	Intraperitoneal injection(ip)	100 mg/kg	2 h and 24 h before ligation	⑫⑬⑭	Ferroptosis, Oxidative Stress, Inflammation
2	Guo et al. (2018)	Male SD rats (200–220 g)	Ligature LAD 30 min, reperfusion 3 h	10/8	Intragastric administration(ig)	25/50/100 mg/kg	Postoperatively once every other day for 4 consecutive weeks	②③⑥⑩⑪⑬⑰⑱	VEGFA/Ang-1, apoptosis suppression, NF-κB
3	Hu et al. (2024)	Male C57BL/6 mice	Ligature LAD60min, reperfusion 24 h	6/6	Intragastric administration(ig)	100 mg/kg	3 consecutive weeks	④⑦⑫⑬⑭	Ferroptosis, VDAC1
4	Qiao et al. (2025)	Male SD rats (230–240 g)	Ligature LAD 30 min, reperfusion	3/3	Intragastric administration(ig)	—	6 consecutive weeks	⑩⑫⑭	14-3-3η-mediated inhibition of ferroptosis, mitochondrial function
5	Yuan et al. (2025)	Male SD rats	Ligature LAD4 5 min, reperfusion 24 h	3/3	Intraperitoneal injection(ip)	100 mg/kg	3 consecutive weeks	④⑦⑩⑫⑭	HES1-mediated, xcessive autophagy, anti-oxidative stress, anti-apoptosis
6	Han et al. (2021)	Male SD rats (220 ± 5g)	Ligature LAD 30 min, reperfusion	3/3	Intravenous injection(iv)	50 mg/kg	20 min after ligation	⑫⑯	LncRNA ANRIL leading to autophagy inhibition
7	Li et al. (2018)	Male SD rats (200–220 g)	Ligature LAD and reperfusion	8/7	Intraperitoneal injection(ip)	5/10 mg/kg	4 h after reperfusion	⑪⑫⑬⑭⑰⑱㉓	AMPK/Akt/GSK-3β/Nrf2 signaling activation
8	Wang. (2005)	Male Wistar rats	Ligature LAD and reperfusion	10/10	Intravenous injection(iv)	100 mg/kg	5 min before ligation and 25 min after ligation	⑧⑪	Apoptosis, modulating Fas/Bcl-2 expression
9	Gao et al. (2006)	Male Wistar rats (200–250 g)	Ligature LAD and reperfusion	11/11	Intravenous injection(iv)	100 mg/kg	5 min before ligation	⑧⑨⑩⑯	Ischemic preconditioning mimicry, apoptosis inhibition
10	Bao et al. (2007)	Male SD rats	Langendorff ischemia 30 min,reperfusion 30 min	10/10	Intragastric administration(ig)	100/200 mg/kg	Before ligation	⑪⑬⑮	Oxidative stress, vascular endothelial function
11	Yun. (2008)	Male SD rats (250–300 g)	Ligature LAD 30 min and reperfusion 30 min	5/5	Intravenous injection(iv)	150 mg/kg	30 min after ligation	⑫⑭	Inflammation (NF-κB), ECM remodeling (MMP-2/TIMP-2)
12	Wang et al. (2008)	Male or female SD rats (250–300 g)	Ligature LAD and reperfusion	10/10	Intravenous injection(iv)	100 mg/kg	1 h before ligation	⑩⑪⑫⑬⑯	Oxidative stress, biological membrane stabilization
13	Ma et al. (2009)	Male SD rats (250∼300 g)	Ligature LAD 30 min, reperfusion 2 h	8/8	Intravenous injection(iv)	2.5 mL/kg	15 min before ligation	①⑤⑨⑩⑮⑳㉑	PI3K/Akt signaling pathway activation, vascular endothelial function improvement
14	Pan et al. (2010)	Male or female SD rats (200–250 g)	Ligature LAD and reperfusion	12/12	Intravenous injection(iv)	20 mg/kg	Before ligation and after reperfusion	⑬⑯	Inflammatory response (ICAM-1mRNA), oxygen free radicals
15	Jia et al. (2010)	Male SD rats (220∼250 g)	Ligature LAD 30 min, reperfusion 3 h	6/6	Intravenous injection(iv)	2/5/10 mg/kg	Before ligation	⑧⑩⑪⑬⑲	Oxidative stress, anti-apoptosis
16	Guo. (2010)	Equal numbers of male and female SD rats (250∼350 g)	Ligature LAD 30 min, reperfusion 3 h	10/10	Intravenous injection(iv)	100 mg/kg	10 min before ligation	⑫⑭	Synergistic antagonism of calcium overload, anti-oxygen free radicals
17	Wu et al. (2011)	Male SD rats (250∼350 g)	Ligature LAD 30 min, reperfusion 2 h	15/15	Intravenous injection(iv)	50 mg/kg	30 min before ligation	⑧	Rho kinase activity, anti-apoptosis, intracellular Ca^2+^ concentration
18	Lin. (2011)	Male SD rats (200–250 g)	Ligature LAD 45 min, reperfusion 2 h	6/6	Intraperitoneal injection(ip)	2.5 mL/kg	15 min or 30 min before ligation	①⑤⑨⑩⑭⑮⑳㉑	PI3K/Akt signaling pathway activation, eNOS phosphorylation
19	Li et al. (2013)	Male Wistar rats (210 ± 10 g)	Ligature LAD 30 min, reperfusion 2 h	10/10	Intravenous injection(iv)	2/5/10 mg/kg	30 min before ligation	⑩⑪⑫⑬⑮⑲	Antioxidative stress
20	Long et al. (2014)	Female SD rats (200-300 g)	Ligature LAD 30 min, reperfusion 4 h	8/8	Intravenous injection(iv)	20 mg/kg	24 h before ligation and postoperatively once every day for 1 week	⑩	TLR4 expression, anti-inflammation
21	Feng et al. (2014)	Male SD rats	Ligature LAD 30 min, reperfusion 2 h	8/8	Intravenous injection(iv)	2.5 mL/kg	15 min before ligation	⑪⑬	Lipid peroxidation, excessive endoplasmic reticulum stress (ERS)
22	Zhou et al. (2016)	Male SD rats (200 ± 20 g)	Ligature LAD and reperfusion	10/10	Intravenous injection(iv)	200 mg/kg	15 min before ligation	⑧	NF-κB signaling pathway, anti-apoptosis (regulation of Bcl-2/Bax)
23	Xuān and Shūxián (2017)	Male SD rats (260∼350 g)	Ligature LAD 30 min, reperfusion 3 h	20/20	Intravenous injection(iv)	5 mg/kg	After ligation	⑧⑩	Antioxidative stress, anti-apoptosis (regulation of Bcl-2/Bax), Akt phosphorylation
24	Li et al. (2017)	Male SD rats (250 ± 20 g)	Ligature LAD 30 min, reperfusion 2 h	10/10	Intravenous injection(iv)	20 mg/kg	5 min after ligaton	⑧⑩	Mitochondrial mPTP opening, Anti-apoptosis,autophagy
25	Liu. (2018)	Male SD rats (200–220 g)	Ligature LAD 30 min, reperfusion 2 h	10/10	Intravenous injection(iv)	20 mg/kg	5 min after ligaton	⑮⑯	Inflammatory response (downregulation of CRP), NO
26	Wang et al. (2020)	Male SD rats (220–250 g)	Ligature LAD 40 min, reperfusion	8/8	Extracorporeal perfusion	10 μmol/L	40 min after ligation	①⑥⑩⑫	Upregulation of Sirt1, FOXO1 acetylation, anti-apoptosis
27	Wang et al. (2021)	Male SD rats (240–260 g)	Ligature LAD 1 h, reperfusion 24 h	6/6	Intragastric administration(ig)	10/30/100 mg/kg	After ligation	②③⑧⑫⑭⑰⑱㉒㉓	TLR4/Myd88/NF-κB pathway, NLRP3 inflammasome activation, anti-inflammation, anti-apoptosis
28	Ma. (2021)	Male SD rats (250–300 g)	Ligature LAD15 min, reperfusion 30 min	8/8	Intraperitoneal injection(ip)	1.6/3.2/6.4 g/kg	1 h before ligation	⑧⑨⑪⑬⑭⑰⑱㉒	MAPK p38/NF-κb p65 pathway, Antioxidative stress, ion channels, Cx43, anti-apoptosis, anti-arrhythmia
29	Zhang. (2023)	Male C57BL/6 mice	Ligature LAD 30 min, reperfusion	6/6	Intraperitoneal injection(ip)	50 mg/kg	Postoperatively once every day for 1 week	④⑦⑧⑩	FUNDC1 expression, mitophagy, anti-apoptosis, mitochondrial function protection

i.g, intragastric administration; i.p, intraperitoneal injection; i.v, intravenous injection; ecp, extracorporeal perfusion; No., number; LAD, left anterior descending coronary artery; SD, Sprague–Dawley; VEGFA, vascular endothelial growth factor A; VDAC1, voltage-dependent anion-selective channel 1; TIMP-2, tissue inhibitor of metalloproteinase 2; TLR4, Toll-like receptor 4; Sirt1, sirtuin 1; PI3K, phosphatidylinositol 3-kinase; Nrf2, nuclear factor erythroid 2-related factor 2; NLRP3, NOD-like receptor family pyrin domain containing 3; NF-κB, nuclear factor kappa-light-chain-enhancer of activated B cells; MyD88, myeloid differentiation primary response 88; mPTP, mitochondrial permeability transition pore; MMP-2, matrix metalloproteinase 2; MAPK, mitogen-activated protein kinase; LncRNA, long non-coding RNA; ICAM-1, intercellular adhesion molecule 1; GSK-3β, glycogen synthase kinase 3 beta; FUNDC1, FUN14 domain containing 1; FOXO1, Forkhead box O1; Fas, factor-associated suicide; ERS, endoplasmic reticulum stress; eNOS, endothelial nitric oxide synthase; ECM, extracellular matrix; CRP, C-reactive protein; Cx43, connexin 43; Bcl-2, B-cell lymphoma-2; Ang-1, angiopoietin-1; AMPK, adenosine monophosphate-activated protein kinase; LVSP, left ventricular systolic pressure; LVIDS, left ventricular internal dimension in systole; LVIDD, left ventricular internal dimension in diastole; LVEF, left ventricular ejection fraction; LVEDP, left ventricular end-diastolic pressure; LVDP, left ventricular diastolic pressure; LVFS, left ventricular fractional shortening; AI, cardiomyocyte apoptosis index; myocardial ischemic size, myocardial infarction size; SOD, superoxide dismutase; LDH, lactate dehydrogenase; MDA, malondialdehyde; CK-MB, creatine kinase-MB; NO, nitric oxide; CK, creatine kinase; TNF-α, tumor necrosis factor-α; IL-6, interleukin-6; GSH, glutathione; + dp/dtmax, maximum rate of left ventricular pressure rise; -dp/dtmax, maximum rate of left ventricular pressure decline; cTn-T, cardiac troponin; TIL-1β, interleukin-1beta.

### 3.3 Methodological quality evaluation

**FIGURE 3:**
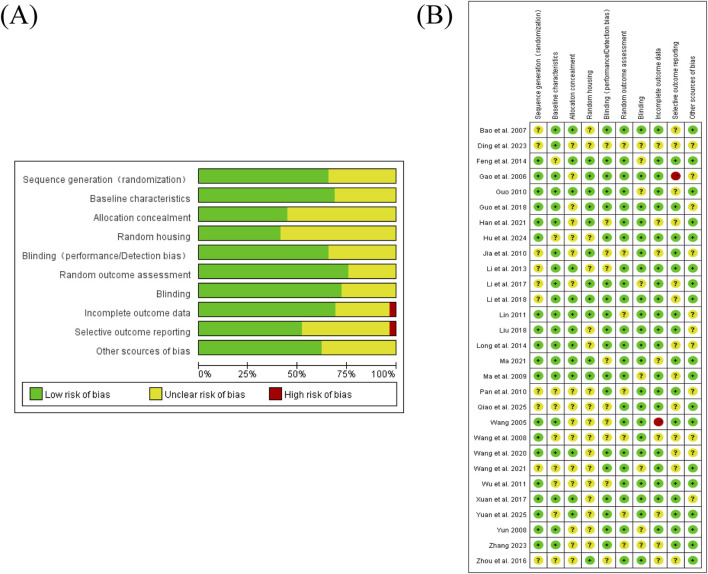
Risk of bias and quality assessment of the included studies: **(A)** risk-of-bias graph; **(B)** risk-of-bias summary.

The SYRCLE risk-of-bias assessments for the included studies revealed notable variations across domains. In sequence generation, most studies demonstrated low risk (Hu et al., 2024; Yuan et al., 2025) or unclear risk (Ding et al., 2023; Qiao et al., 2025). Baseline characteristics were generally well controlled, with the majority rated as low risk (Ding et al., 2023; Guo et al., 2018), and only a few as unclear risk (Hu et al., 2024). However, incomplete outcome data indicated a higher risk, with studies such as Wang (2005) rated as high risk, whereas several other studies were assessed as having an unclear risk. Random housing was predominantly unclear, although some studies showed low risk (Guo et al., 2018; Li et al., 2018). Performance/detection blinding varied a little, with several studies rated as low risk (Guo et al., 2018; Hu et al., 2024; Bao et al., 2007; Feng et al., 2014). Selective outcome reporting was predominantly assessed as low risk, with one study rated as high risk (Gao et al., 2006). Other sources of bias were generally well managed and were predominantly assessed as low risk (the details are shown in Table 2; Figure 3).

**TABLE 2:** Risk of bias.

No.	Study	Year	Sequence generation (randomization)	Baseline characteristics	Allocation concealment	Random housing	Blinding (performance/detection bias)	Random outcome assessment	Blinding	Incomplete outcome data	Selective outcome reporting	Other sources of bias
1	Ding et al.	2023	Unclear risk	Low risk	Unclear risk	Unclear risk	Unclear risk	Unclear risk	Unclear risk	Unclear risk	Unclear risk	Unclear risk
2	Guo et al.	2018	Low risk	Low risk	Unclear risk	Low risk	Low risk	Low risk	Low risk	Low risk	Low risk	Unclear risk
3	Hu et al.	2024	Low risk	Unclear risk	Unclear risk	Unclear risk	Low risk	Low risk	Low risk	Low risk	Low risk	Low risk
4	Qiao et al.	2025	Unclear risk	Unclear risk	Unclear risk	Unclear risk	Unclear risk	Low risk	Low risk	Low risk	Unclear risk	Low risk
5	Yuan et al.	2025	Low risk	Unclear risk	Low risk	Unclear risk	Low risk	Unclear risk	Low risk	Unclear risk	Low risk	Low risk
6	Han et al.	2021	Low risk	Low risk	Unclear risk	Low risk	Unclear risk	Low risk	Low risk	Unclear risk	Unclear risk	Low risk
7	Li et al.	2018	Unclear risk	Low risk	Low risk	Low risk	Low risk	Low risk	Low risk	Low risk	Unclear risk	Low risk
8	Wang	2005	Low risk	Low risk	Unclear risk	Unclear risk	Unclear risk	Low risk	Low risk	High risk	Low risk	Low risk
9	Gao et al.	2006	Low risk	Low risk	Unclear risk	Low risk	Low risk	Low risk	Low risk	Low risk	High risk	Unclear risk
10	Bao et al.	2007	Unclear risk	Low risk	Low risk	Unclear risk	Low risk	Low risk	Low risk	Low risk	Unclear risk	Low risk
11	Yun	2008	Low risk	Low risk	Unclear risk	Unclear risk	Low risk	Low risk	Unclear risk	Low risk	Low risk	Low risk
12	Wang et al.	2008	Low risk	Unclear risk	Unclear risk	Unclear risk	Unclear risk	Unclear risk	Low risk	Unclear risk	Unclear risk	Unclear risk
13	Ma et al.	2009	Low risk	Low risk	Low risk	Low risk	Low risk	Low risk	Unclear risk	Low risk	Unclear risk	Low risk
14	Pan et al.	2010	Unclear risk	Unclear risk	Unclear risk	Unclear risk	Low risk	Unclear risk	Low risk	Low risk	Low risk	Unclear risk
15	Jia et al.	2010	Unclear risk	Low risk	Unclear risk	Low risk	Unclear risk	Unclear risk	Low risk	Unclear risk	Low risk	Unclear risk
16	Guo	2010	Low risk	Low risk	Low risk	Low risk	Low risk	Low risk	Unclear risk	Low risk	Unclear risk	Low risk
17	Wu et al.	2011	Low risk	Unclear risk	Unclear risk	Unclear risk	Unclear risk	Low risk	Low risk	Low risk	Low risk	Low risk
18	Lin	2011	Low risk	Low risk	Low risk	Low risk	Low risk	Unclear risk	Low risk	Low risk	Low risk	Unclear risk
19	Li et al.	2013	Unclear risk	Low risk	Low risk	Unclear risk	Unclear risk	Low risk	Low risk	Low risk	Low risk	Low risk
20	Long et al.	2014	Low risk	Low risk	Low risk	Unclear risk	Low risk	Low risk	Low risk	Low risk	Unclear risk	Unclear risk
21	Feng et al.	2014	Low risk	Unclear risk	Low risk	Low risk	Low risk	Low risk	Unclear risk	Low risk	Low risk	Low risk
22	Zhou et al.	2016	Unclear risk	Unclear risk	Unclear risk	Low risk	Unclear risk	Low risk	Low risk	Unclear risk	Unclear risk	Low risk
23	Xuan et al.	2017	Low risk	Low risk	Low risk	Unclear risk	Low risk	Low risk	Low risk	Low risk	Low risk	Unclear risk
24	Li et al.	2017	Unclear risk	Low risk	Unclear risk	Low risk	Low risk	Low risk	Unclear risk	Low risk	Unclear risk	Low risk
25	Liu	2018	Low risk	Low risk	Low risk	Unclear risk	Low risk	Low risk	Low risk	Low risk	Low risk	Unclear risk
26	Wang et al.	2020	Low risk	Low risk	Low risk	Unclear risk	Low risk	Low risk	Low risk	Low risk	Unclear risk	Unclear risk
27	Wang et al.	2021	Unclear risk	Unclear risk	Unclear risk	Unclear risk	Low risk	Low risk	Unclear risk	Low risk	Unclear risk	Low risk
28	Ma	2021	Low risk	Low risk	Low risk	Low risk	Unclear risk	Low risk	Low risk	Unclear risk	Low risk	Low risk
29	Zhang	2023	Low risk	Low risk	Unclear risk	Unclear risk	Low risk	Unclear risk	Unclear risk	Unclear risk	Low risk	Low risk

### 3.4 Meta-analysis results

#### 3.4.1 Myocardial injury markers

This analysis included four indicators related to myocardial injury markers reported across the 29 included studies: cardiac troponin T (cTn-T), creatine kinase (CK), creatine kinase-MB (CK-MB), and lactate dehydrogenase (LDH).

**FIGURE 4:**
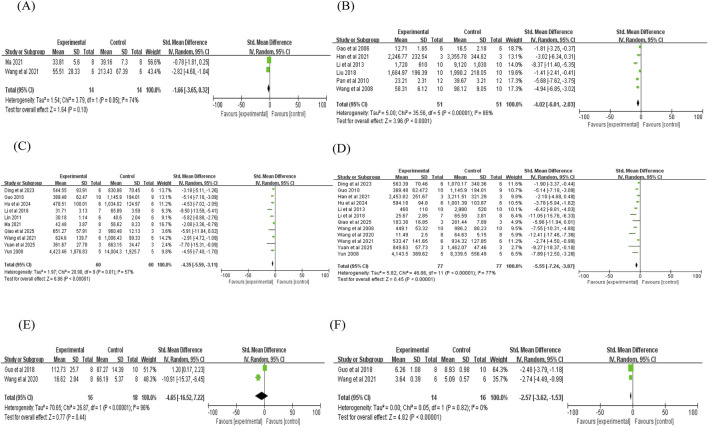
Forest plot of cTnT, CK, CK-MB, LDH, LVDP, and LVIDs: **(A)** forest plot of cTnT; **(B)** forest plot of CK; **(C)** forest plot of CK-MB; **(D)** forest plot of LDH; **(E)** forest plot of LVDP; **(F)** forest plot of LVIDs.

Two studies reported that puerarin was associated with a reduction in cTn-T levels compared with controls (n = 2; SMD = −1.66; 95% CI: −3.65 to 0.32; heterogeneity: I^2^ = 74%; P = 0.05; Figure 4A). However, given that this effect was based on only two studies with small sample sizes and the 95% CI crossed zero, these findings should be interpreted cautiously, as they may be influenced by small-study bias and do not indicate a definitive therapeutic effect. In six studies, puerarin indicated a potential reduction in CK levels (n = 6; SMD = −4.02; 95% CI: −6.01 to −2.03; heterogeneity: I^2^ = 86%; P < 0.00001; Figure 4B). Similarly, 10 studies showed a trend toward lower CK-MB levels compared with controls (n = 10; SMD = −4.35; 95% CI: −5.59 to −3.11; heterogeneity: I^2^ = 57%; P = 0.01; Figure 4C). Furthermore, 12 studies demonstrated lower LDH levels with puerarin intervention (n = 12; SMD = −5.55; 95% CI: −7.24 to −3.87; heterogeneity: I^2^ = 77%; P < 0.00001; Figure 4D). The justification for performing meta-analytic pooling despite the observed heterogeneity is as follows: all studies focused on the effect of puerarin on MIRI and utilized consistent outcome indicators (myocardial injury markers), thereby avoiding fundamental methodological differences that would preclude pooling; subgroup analysis was further conducted to explore the potential sources of heterogeneity.

#### 3.4.2 Hemodynamic parameters

This analysis included nine indicators related to hemodynamic parameters reported across the 29 included studies: left ventricular diastolic pressure (LVDP), left ventricular internal dimension in systole (LVIDs), left ventricular end-diastolic pressure (LVEDP), left ventricular internal dimension in diastole (LVIDd), left ventricular systolic pressure (LVSP), left ventricular ejection fraction (LVEF), left ventricular fractional shortening (LVFS), the maximum rate of left ventricular pressure rise (+dp/dtmax), and the maximum rate of left ventricular pressure decline (-dp/dtmax).

**FIGURE 5:**
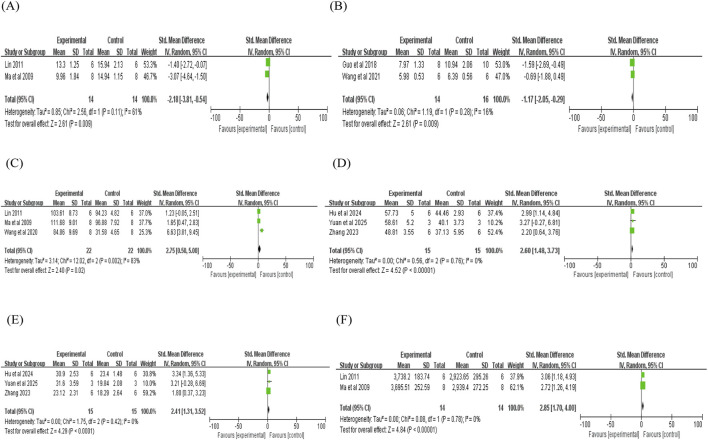
Forest plot of LVEDP, LVIDd, LVSP, LVEF, LVFS, and +dp/dtmax: **(A)** forest plot of LVEDP; **(B)** forest plot of LVIDd; **(C)** forest plot of LVSP; **(D)** forest plot of LVEF; **(E)** forest plot of LVFS; **(F)** forest plot of +dp/dtmax.

**FIGURE 6:**
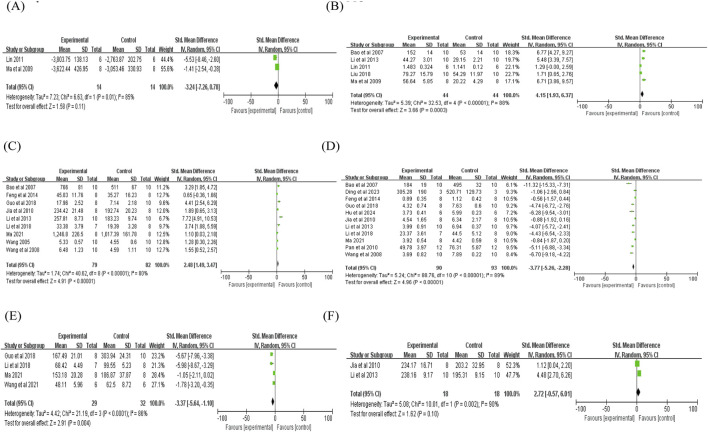
Forest plot of -dp/dtmax, NO, SOD, MDA, TNF-α, and GSH: **(A)** forest plot of -dp/dtmax; **(B)** forest plot of NO; **(C)** forest plot of SOD; **(D)** forest plot of MDA; **(E)** forest plot of TNF-α; **(F)** forest plot of GSH.

Two studies reported that puerarin reduced LVDP (n = 2; SMD = −4.65; 95% CI: −16.52 to 7.22; heterogeneity: I^2^ = 96%; P < 0.00001; Figure 4E). Regarding LVIDs, two studies demonstrated a significant reduction compared with controls (n = 2; SMD = −2.57; 95% CI: −3.62 to −1.53; heterogeneity: I^2^ = 0%; P = 0.82; Figure 4F). Similarly, a decrease in LVEDP was observed in two studies (n = 2; SMD = −2.18; 95% CI: −3.81 to −0.54; heterogeneity: I^2^ = 61%; P = 0.11; Figure 5A). Additionally, two studies indicated that puerarin reduced LVIDd (n = 2; SMD = −1.17; 95% CI: −2.05 to −0.29; heterogeneity: I^2^ = 16%; P = 0.28; Figure 5B). In terms of systolic function, three studies demonstrated that puerarin increased LVSP (n = 3; SMD = 1.46; 95% CI: 0.59 to 2.32; heterogeneity: I^2^ = 0%; P = 0.64; Figure 5C). Moreover, improvements were observed in LVEF across three studies (n = 3; SMD = 2.60; 95% CI: 1.48 to 3.73; heterogeneity: I^2^ = 0%; P = 0.76; Figure 5D), along with that in LVFS (n = 3; SMD = 2.41; 95% CI: 1.31 to 3.52; heterogeneity: I^2^ = 0%; P = 0.42; Figure 5E). Two studies reported that puerarin increased the maximum rate of +dp/dtmax compared with controls (n = 2; SMD = 2.85; 95% CI: 1.70 to 4.00; heterogeneity: I^2^ = 0%; P = 0.78; Figure 5F). Another two studies reported that puerarin reduced the -dp/dtmax (n = 2; SMD = −3.24; 95% CI: −7.26 to 0.78; heterogeneity: I^2^ = 85%; P = 0.01; Figure 6A). For hemodynamic parameters, many key effects were derived from only 2–3 studies and showed large effect sizes (absolute SMD >1.4), indicating the potential presence of small-study bias.

#### 3.4.3 Oxidative stress markers

The analysis included three indicators related to oxidative stress markers reported across the 29 included studies: nitric oxide (NO), superoxide dismutase (SOD), and malondialdehyde (MDA). Five studies showed a tendency toward elevated NO levels after puerarin treatment (n = 5; SMD = 4.15; 95% CI: 1.93 to 6.37; heterogeneity: I^2^ = 88%; P < 0.00001; Figure 6B). In addition, nine studies indicated that puerarin elevated SOD levels (n = 9; SMD = 2.48; 95% CI: 1.49 to 3.47; heterogeneity: I^2^ = 80%; P < 0.00001; Figure 6C). Moreover, analysis of 11 studies demonstrated a decrease in MDA levels (n = 11; SMD = −3.77; 95% CI: −5.26 to −2.28; heterogeneity: I^2^ = 89%; P < 0.00001; Figure 6D). Some studies lacked adequate control for confounding factors (e.g., baseline oxidative stress levels), which may have contributed to heterogeneity.

#### 3.4.4 Inflammatory cytokines analyzed

**FIGURE 7:**
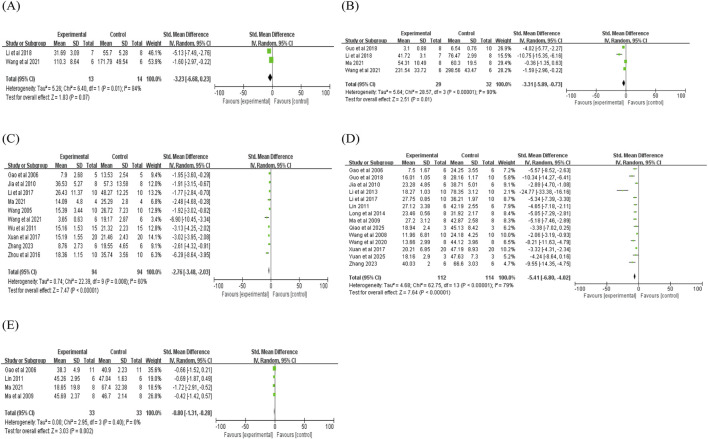
Forest plot of IL-1β, IL-6, AI (cardiomyocyte apoptosis index), myocardial infarction size, and myocardial ischemic size: **(A)** forest plot of IL-1β; **(B)** forest plot of IL-6; **(C)** forest plot of AI; **(D)** forest plot of myocardial infarction size; **(E)** forest plot of myocardial ischemic size.

The analysis included four indicators related to inflammatory cytokines, which were reported across the 29 included studies: tumor necrosis factor-α (TNF-α), glutathione (GSH), interleukin-1 beta (IL-1β), and interleukin-6 (IL-6). Four studies indicated a decrease in TNF-α levels with puerarin (n = 4; SMD = −3.37; 95% CI: −5.64 to −1.10; heterogeneity: I^2^ = 86%; P < 0.00001; Figure 6E). Two studies reported a numerical increase in GSH levels following puerarin treatment, but the difference was not statistically significant (n = 2; SMD = 2.72; 95% CI: −0.57 to 6.01; heterogeneity: I^2^ = 90%; P = 0.002; Figure 6F). Two studies reported a numerical decrease in IL-1β levels with puerarin intervention, but the difference did not reach statistical significance (n = 2; SMD = −3.23; 95% CI: −6.68 to 0.23; heterogeneity: I^2^ = 84%; P = 0.01; Figure 7A). Finally, four studies showed lower interleukin-6 (IL-6) levels (n = 4; SMD = −3.31; 95% CI: −5.89 to −0.73; heterogeneity: I^2^ = 90%; P < 0.00001; Figure 7B). Heterogeneity (I^2^ = 84%–90%) was present for all inflammatory cytokines, which may be attributed to variations in puerarin dosage and modeling duration.

#### 3.4.5 Apoptosis-related indices and cardiac tissue structure

This analysis included three indicators: cardiomyocyte apoptosis index (AI), myocardial infarction size, and myocardial ischemic size. A total of 10 studies reported that puerarin reduced the cardiomyocyte AI (n = 10; SMD = −2.76; 95% CI: −3.48 to −2.03; heterogeneity: I^2^ = 60%; P = 0.008; Figure 7C). A pooled analysis of 14 studies demonstrated a decrease in myocardial infarction size (n = 14; SMD = −5.41; 95% CI: −6.80 to −4.02; heterogeneity: I^2^ = 79%; P < 0.00001; Figure 7D). Additionally, in four studies, puerarin was associated with a reduction in myocardial ischemic size (n = 4; SMD = −0.80; 95% CI: −1.31 to −0.28; heterogeneity: I^2^ = 0%; P = 0.40; Figure 7E). The justification for pooling is the consistency of outcome definitions and the focus on MIRI. Heterogeneity was potentially due to publication bias and methodological differences in myocardial infarction size measurement (e.g., TTC staining vs. histological scoring). For cardiomyocyte AI, the moderate heterogeneity may be explained by differences in the apoptosis detection methods. Some studies lacked blinding of histological assessors, which may have introduced bias into measurements of the apoptosis index and myocardial infarction size.

### 3.5 Subgroup analysis

Subgroup analyses were performed to explore potential sources of the moderate-to-high heterogeneity observed in the main meta-analysis, focusing on three key covariates: animal body weight, puerarin dosage, and administration route. These analyses were prespecified to address potential variability between studies.

**FIGURE 8:**
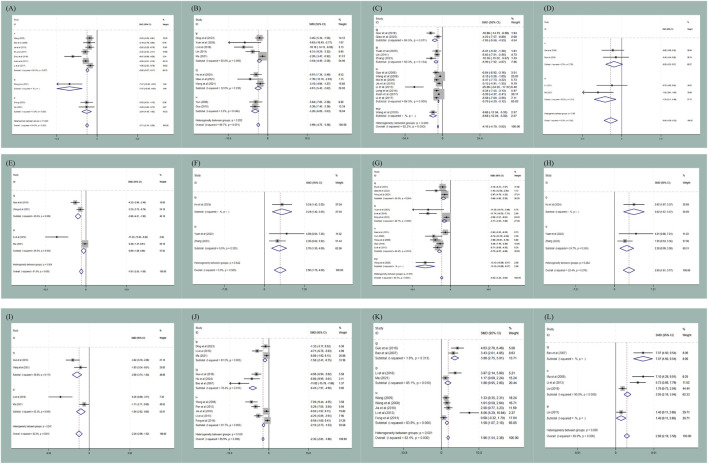
Subgroup analysis of administration route: **(A)** subgroup analysis of administration route to AI; **(B)** subgroup analysis of administration route to CK-MB; **(C)** subgroup analysis of administration route to myocardial infarction size; **(D)** subgroup analysis of administration route to myocardial ischemic size; **(E)** subgroup analysis of administration route to IL-6; **(F)** subgroup analysis of administration route to LVEF; **(G)** subgroup analysis of administration route to LDH; **(H)** subgroup analysis of administration route to LVFS; **(I)** subgroup analysis of administration route to TNF-α; **(J)** subgroup analysis of administration route to MDA; **(K)** subgroup analysis of administration route to SOD; **(L)** subgroup analysis of administration route to NO.

In the administration route subgroup, intragastric administration was associated with greater reductions in IL-6 levels [SMD = −3.08 vs. −0.89, P (between) = 0.004] and MDA levels [SMD = −6.43 vs. −1.58 vs. −2.13, P (between) <0.001], whereas extracorporeal perfusion administration exhibited the most pronounced effects on myocardial infarction size [P (between) < 0.001]. These findings indicate that the administration route may be a potential source of heterogeneity for these endpoints; however, the effect sizes for intragastric administration (especially for MDA, SMD = −6.43) remain large, indicating that publication bias or small-study bias may still be present (detailed results are provided in Figure 8).

**FIGURE 9:**
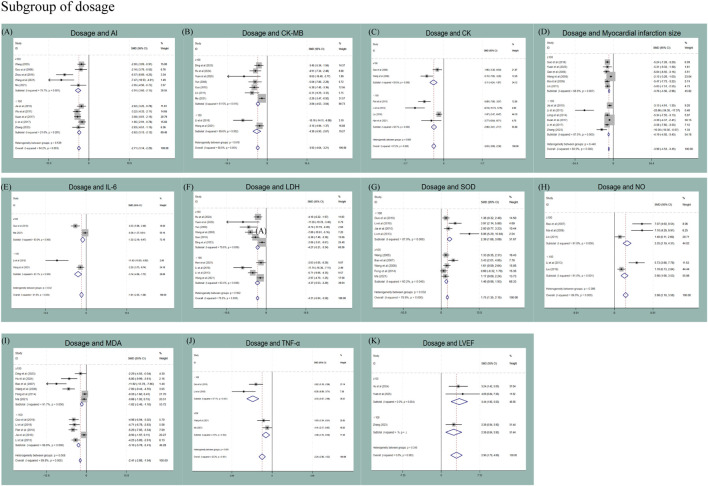
Subgroup analysis of dosage subgroup: **(A)** subgroup analysis of dosage to AI; **(B)** subgroup analysis of dosage to CK-MB; **(C)** subgroup analysis of dosage to CK; **(D)** subgroup analysis of dosage to myocardial infarction size; **(E)** subgroup analysis of dosage to IL-6; **(F)** subgroup analysis of dosage to LDH; **(G)** subgroup analysis of dosage to SOD; **(H)** subgroup analysis of dosage to NO; **(I)** subgroup analysis of dosage to MDA; **(J)** subgroup analysis of dosage to TNF-α; **(K)** subgroup analysis of dosage to LVEF.

In the dosage subgroup, lower puerarin dosage (<100 mg/kg) may produce a decrease in MDA levels [SMD = −3.10 vs. −1.82, P (between) = 0.008], whereas higher dosage (≥100 mg/kg) showed an improvement on LVFS (SMD = 3.72). Dosage may thus contribute to heterogeneity for MDA and LVFS; however, the small number of studies in each dosage stratum may influence the reliability of these findings (detailed results are provided in Figure 9).

**FIGURE 10:**
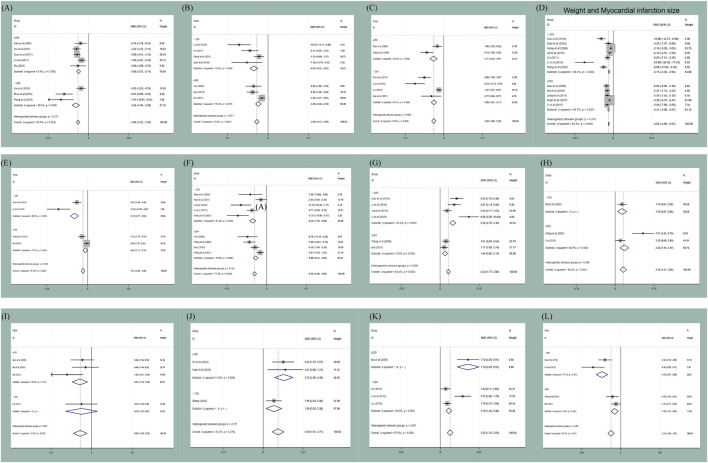
Subgroup analysis of body weight: **(A)** subgroup analysis of body weight to AI; **(B)** subgroup analysis of body weight to CK-MB; **(C)** subgroup analysis of body weight to CK; **(D)** subgroup analysis of body weight to myocardial infarction size; **(E)** subgroup analysis of body weight to IL-6; **(F)** subgroup analysis of body weight to LDH; **(G)** subgroup analysis of body weight to SOD; **(H)** subgroup analysis of body weight to LVSP; **(I)** subgroup analysis of body weight to myocardial ischemic size; **(J)** subgroup analysis of body weight to LVFS; **(K)** subgroup analysis of body weight to NO; **(L)** subgroup analysis of body weight to TNF-α.

Regarding body weight, animals weighing <250 g exhibited an association with IL-6 reduction [P (between) < 0.001], whereas animals weighing ≥250 g showed more effect on NO [P (between) = 0.001]. This may be related to variations in ischemia duration, reperfusion time, and specific surgical techniques (detailed results are provided in Figure 10).

### 3.6 Publication bias

**FIGURE 11:**
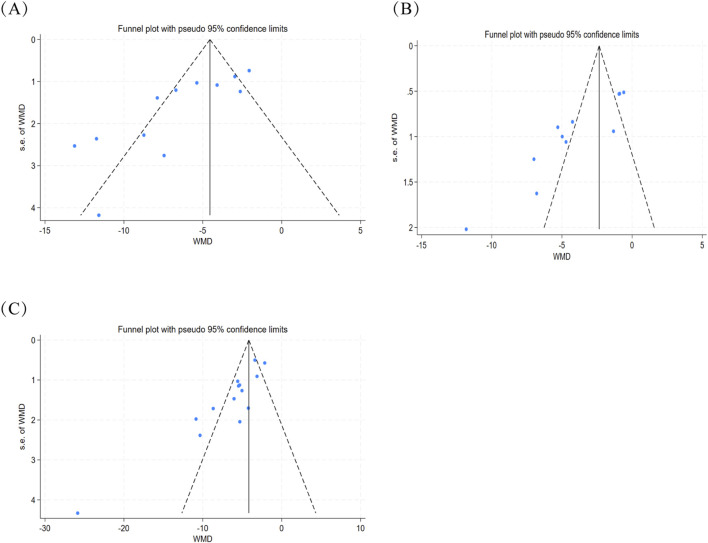
Publication bias of myocardial infarction size, LDH, or MDA: **(A)** publication bias of LDH; **(B)** publication bias of MDA; **(C)** publication bias of myocardial infarction size.

Publication bias arises when dissemination of research findings is contingent upon statistical significance or effect direction, thereby inflating estimated intervention effects. Three indicators, namely, MDA, LDH, and myocardial infarction size, were reported in more than 10 studies. Therefore, Egger’s test was conducted to assess potential publication bias. For MDA, Egger’s test indicated publication bias (P < 0.005; P = 0.000). Similarly, Egger’s test for LDH and myocardial infarction size also indicated a publication bias (P < 0.005; P = 0.001; P < 0.005; P = 0.000) (Figure 11). These results indicate the presence of some publication bias in these indicators. Visual inspection of funnel plots revealed asymmetry for outcomes (Supplementary Figure S5), and Egger’s test confirmed statistically significant asymmetry for the endpoints. Both methods provided evidence consistent with selective reporting. This pattern likely reflects the preclinical nature of our evidence base: animal intervention studies exhibit a well-documented propensity toward preferential publication of statistically favorable results, a phenomenon that may have attenuated representation of null or negative findings in the available literature. We therefore performed trim-and-fill analyses as sensitivity corrections for the three outcomes in which bias was indicated: no missing studies were imputed for myocardial infarction size, LDH, or MDA (zero studies trimmed/filled; estimates unchanged) (Supplementary Figure S1).

### 3.7 Sensitivity analysis

Meanwhile, sensitivity analysis was conducted for three indicators (MDA, LDH, and myocardial infarction size).

In the assessment of myocardial infarction size, the studies by Wang et al. (2008) and Xuān and Shūxián (2017) exert influence on heterogeneity. When these two articles are excluded, the effect size shows fluctuations. The direction and statistical significance of the main effects remained consistent, as illustrated in the myocardial infarction size sensitivity plot.

Regarding MDA levels, the reports of Jia et al. (2010), Feng et al. (2014), and Ma (2021) affect the heterogeneity. Removal of these three studies’ results led to fluctuations in the effect size, and yet, the direction and statistical significance of the main effects remained constant, as shown in the MDA sensitivity plot.

For LDH, the article by Ding et al. (2023) has an impact on heterogeneity. Removing this publication leads to fluctuations in the direction and statistical significance, but the main effects were nevertheless unchanged, as depicted in the LDH sensitivity plot.

**FIGURE 12:**
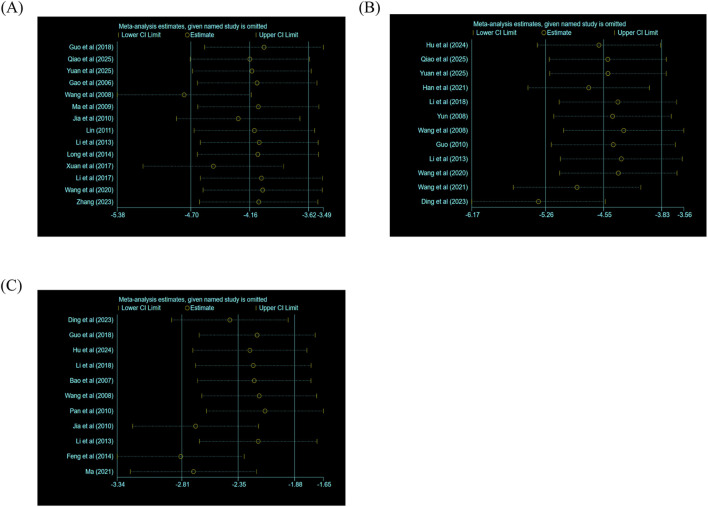
Sensitivity analysis of myocardial infarction size, LDH, or MDA: **(A)** sensitivity analysis of myocardial infarction size; **(B)** sensitivity analysis of LDH; **(C)** sensitivity analysis of MDA.

These results collectively indicate that the robustness of the pooled results for these three indicators may have some limitations; it is probably caused by the gap between the highly controlled laboratory environment and complex clinical practice (Figure 12).

### 3.8 Valuation of evidence quality

A total of 11 outcomes were selected to assess the evidence quality using the GRADE tool, which classifies evidence into high, moderate, low, or very low based on five domains: risk-of-bias, inconsistency, indirectness, imprecision, and publication bias. The presence of any of these limitations reduces the certainty of the evidence. In this analysis, the evidence for the effects of puerarin on MIRI showed varying quality across different outcomes. High-quality evidence was observed for three critical outcomes: AI, CK-MB, and myocardial ischemic size. For four other critical outcomes—LDH, MDA, myocardial infarction size, and SOD—the evidence was rated as of moderate quality; the main limitation was the risk of bias or inconsistency. In contrast, the remaining outcomes—GSH, LVDP, NO, and TNF-α—were supported by low-quality evidence. The limitations contributing to this lower certainty included risk of bias combined with imprecision, risk of bias plus inconsistency, inconsistency and imprecision, and risk of bias with suspected publication bias. In brief, the critical outcomes demonstrated favorable effects of puerarin (either reduced harmful markers or increased protective factors), whereas the important outcomes showed inconsistent directional effects, which may be influenced by the low certainty of evidence (Supplementary Figure S3).

## 4 Discussion

Animal studies constitute the foundational step prior to clinical trials. They enable the evaluation of intervention safety, the assessment of dose–response relationships, and preliminary efficacy judgments, thereby generating critical evidence for informed decision-making regarding the advancement of therapeutic strategies or novel drugs into human trials.

This study comprehensively evaluated the preclinical evidence regarding the cardioprotective effects of puerarin against MIRI. By synthesizing data from 29 animal studies, our analysis demonstrates that puerarin administration is associated with attenuating MIRI, as evidenced by improved cardiac function, reduced myocardial infarction size, suppressed oxidative stress and inflammation, and decreased cardiomyocyte apoptosis. The improvements in LVEF and LVFS indicate enhanced systolic function. In addition, it reduced LVIDd, LVIDs, and LVEDP, indicating ameliorated ventricular remodeling and diastolic dysfunction. Notably, puerarin treatment was associated with a substantial reduction in myocardial infarction size, which is a critical determinant of long-term prognosis. These results confirmed that puerarin not only preserves viable myocardium but also promotes functional recovery following the acute phase I/R.

**FIGURE 13:**
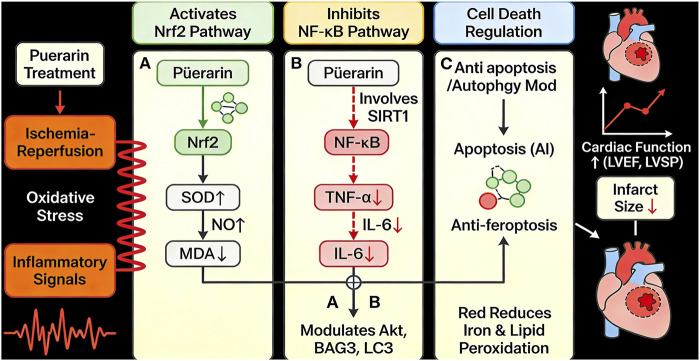
Effects of puerarin on various signaling pathways: **(A)** Nrf2 pathway; **(B)** NF-κB pathway; **(C)** anti-apoptosis and autophagy modulation.

This meta-analysis quantitatively showed that puerarin mitigates MIRI through the modulation of key pathological pathways that were previously outlined. The pooled reduction in oxidative stress markers (MDA SMD = −3.77) alongside increased activity of antioxidant defenses (SOD SMD = 2.48; NO SMD = 4.15) provides quantitative support for its role in attenuating oxidative damage, a primary driver of reperfusion injury (Zweier and Talukder, 2006). Similarly, the decrease in pro-inflammatory cytokines (TNF-α SMD = −3.37; IL-6 SMD = −3.31) substantiate its anti-inflammatory effects at a pooled effect size level. Autophagy acts as a “double-edged sword” in MIRI, where moderate autophagy may be protective, whereas excessive autophagy leads to damage. The existing research findings on the regulatory effect of puerarin on autophagy appear to be inconsistent, which may be attributed to differences in study models, intervention timing, and detection time-points. The reduction in the cardiomyocyte apoptosis index (AI SMD = −2.76) is consistent with studies reporting that puerarin decreases the LC3-II/LC3-I ratio via activation of the Akt pathway (Tang et al., 2017). Thus, the present meta-analysis summaries by providing pooled quantitative estimates that evaluate the physiological relevance of these pathways in the observed cardioprotection, thereby strengthening the evidence base for its multi-target action (Figure 13).

The biomarkers commonly used to assess myocardial injury and infarction include cTn-I, cTn-T, CK, CK-MB, and LDH, which differ in sensitivity and specificity. Following myocardial ischemia and hypoxia-induced degeneration and necrosis, blood levels of cTn-T begin to rise within 4 h–12 h and remain elevated for 4–10 days (Zhang et al., 2024). Due to its extended half-life, high myocardial concentration, and elevated sensitivity, cTn is regarded as the standard biomarker for myocardial injury. In this study, puerarin was associated with reduced levels of cTn-T, CK, CK-MB, and LDH, indicating attenuation of myocardial damage.

Subgroup analysis was performed on CK-MB, myocardial infarction size, LDH, LVDP, and other indicators according to the route of administration. The results showed that the route of administration had an impact on the heterogeneity of the study, which may be due to the differences in drug absorption and availability. However, this is not the only factor to assess the sources of heterogeneity; heterogeneity may also be related to differences in dose, weight, mode of modeling, ligation, and reperfusion time. In this meta-analysis, several studies (literature) grouped the dose of puerarin into low, medium, and high doses (Guo et al., 2018; Li et al., 2013; Jia et al., 2010; Wang et al., 2021; Ma, 2021). The results of these studies indicated that puerarin exhibited a gradient effect at different doses, with the high-dose group demonstrating the greatest therapeutic effect. We extracted the high-dose group data from these studies for statistical analysis.

### 4.1 Advantages

The preclinical evidence summarized in this review indicates that puerarin may exert cardioprotective effects through a broad spectrum of pharmacological properties. In recent years, growing evidence has highlighted its potential in mitigating MIRI. The mechanisms underlying its protective actions are multifaceted, involving modulation of oxidative stress, apoptosis, autophagy, ferroptosis, and pyroptosis, which collectively indicate a pleiotropic mode of action (Shi et al., 2002). Although the chemical structure of puerarin poses challenges to its administration and bioavailability, these also drive innovative research into formulation strategies and structural derivatives. As mentioned above, clinically, puerarin has been utilized in the management of coronary heart disease, retinal vascular occlusion, and sudden deafness (Meng et al., 2022), supporting its translational relevance (Xie et al., 2018). Currently, the existing clinical applications provide a foundational basis for its assessment in the treatment of MIRI. The complexity of its mechanisms, involving multiple cellular signaling pathways, presents an opportunity for deeper mechanistic exploration. Given the multitude of cellular signaling pathways involved, future studies would further analyze the mechanisms of puerarin in MIRI, develop its derivatives, and improve techniques to enhance its bioavailability, thereby providing new strategies and evidence for MIRI treatment.

### 4.2 Limitations

1. The aforementioned studies have focused on the pharmacological effects of puerarin based on animal and *in vitro* experimental reports. Therefore, well-designed, multicenter, large-sample, randomized controlled trials are required to evaluate the efficacy and adverse effects of puerarin in the treatment of ischemic cardiomyopathy. Additionally, as a potential therapeutic agent with significant prospects for cardiovascular diseases, further research utilizing metabolomics, proteomics, genomics, and network pharmacology should be conducted to elucidate its pharmacological activity and molecular mechanisms. This will pave the way for the future development of more effective puerarin formulations for the prevention and treatment of cardiovascular diseases.
2. The predominant use of male animals in the included studies restricts the generalizability of our findings to both sexes. This approach does not address potential sex-based differences. Therefore, caution is required when translating these results to clinical contexts.
3. Although we observed a consistent trend favoring puerarin for most outcome measures, the I^2^ values for many pooled estimates exceeded 75%, indicating an inconsistency. The likely sources are multifactorial: (1) variability in animal models and surgical procedures: although all studies utilized myocardial I/R models, there were differences in ischemia duration (30 min–60 min), reperfusion time (2 h–24 h), and specific surgical techniques. (2) Widely varying doses (2.5 mg/kg to 6.4 g/kg), timings (pre- or post-ischemia), and treatment durations. (3) Differences in measurement methods and time-points: techniques for assaying biomarkers and assessing cardiac function were not uniform. (4) For certain outcome measures (such as LVEF and LVFS), the point estimates of the pooled standardized mean difference (SMD) were derived from only a small number of studies. Therefore, they should be interpreted with caution, as they may be unstable and more susceptible to exaggeration. However, following the potentially relevant subgroup analyses, we performed meta-regression on the corresponding subgroups, which indicated that the investigated subgroups were not significant sources of heterogeneity for the related outcome measures. For instance, in the meta-regression analysis on the MDA outcome measure, the route of administration (p = 0.038, p < 0.05) as a covariate had an influence on the results, whereas dosage (p = 0.887, p > 0.05) as a covariate had a relatively minor effect, and the body weight subgroup was not analyzed in the indicators as there were fewer than ten studies. Many subgroups contained few studies for robust comparison, and we were unable to perform meta-regression for outcomes due to incomplete reporting of covariates. Consequently, the results of this synthesis may be viewed as evidence of a beneficial trend across diverse experimental settings (Supplementary Figure S2).
4. Risk-of-bias assessment identified concerns across several domains that threaten internal validity. Among the 29 included studies, randomization procedures were adequately described in 19 (65.5%) and allocation concealment in 13 (44.8%). Several studies lacked blinding of investigators and outcome assessors. Deficiencies in randomization and blinding are established sources of selection and detection bias in animal studies, which are known to inflate treatment effect estimates—inadequate randomization may overestimate effects by approximately 30%, and lack of blinding further exaggerates differences, particularly for subjective outcomes such as histological infarction size. Therefore, the pooled effect sizes reported here should be interpreted with caution. Moreover, none of the studies reported *a priori* sample size or power calculations; underpowered experiments may increase uncertainty and contribute to inflated effect estimates.

### 4.3 Clinical relevance and translational considerations

The multi-mechanistic profile and consistent preclinical efficacy of puerarin demonstrated in this meta-analysis highlight its potential translational relevance for MIRI. Clinically, MIRI most frequently complicates the management of acute ST-segment elevation myocardial infarction (STEMI) following rapid revascularization via primary percutaneous coronary intervention (PCI). Although timely reperfusion is essential for limiting the myocardial infarction size, consequent reperfusion injury itself contributes significantly to the final myocardial damage, and no specific therapy is yet routinely used to mitigate this component in clinical practice.

Mechanistically, puerarin appears to simultaneously attenuate several core pathological cascades activated during reperfusion—including oxidative stress, inflammatory cytokine release, and various forms of regulated cell death (apoptosis and ferroptosis). This pleiotropic action differs from the primary mechanisms of standard pharmacological agents used in acute coronary syndromes. For instance, antiplatelet agents and anticoagulants target the thrombotic process, beta-blockers reduce myocardial oxygen demand, and statins exert pleiotropic effects over a longer time frame. Puerarin’s direct targeting of reperfusion-associated cellular injury could therefore represent a complementary strategy aimed specifically at the ischemia–reperfusion interface rather than competing with existing therapies.

However, critical limitations in the preclinical evidence must be acknowledged. The included studies predominantly used healthy, young rodents without common clinical comorbidities such as diabetes, hypertension, or atherosclerosis. Substantial variability existed in experimental protocols—including ischemia duration, reperfusion time, anesthetic agents, puerarin dosage, and administration timing. Notably, most studies utilized pretreatment regimens, which have limited clinical applicability in the setting of unexpected acute myocardial infarction. Evidence supporting post-ischemic or therapeutic administration after reperfusion onset remains sparse. Thus, although the pooled results suggest a robust beneficial trend, the construct validity and direct translatability of these findings are constrained.

In conclusion, the current meta-analysis provides systematic preclinical evidence supporting puerarin’s cardioprotective potential via multi-target mechanisms. Future translational research should prioritize studies using clinically relevant models (e.g., aged animals with comorbidities), standardized ischemia–reperfusion protocols, and administration windows that mimic feasible clinical scenarios (e.g., at reperfusion or shortly thereafter). Such work is necessary to clarify whether puerarin or its optimized derivatives could eventually be developed as an adjunctive therapy to reperfusion in patients at high risk for MIRI.

## 5 Conclusion

In summary, this meta-analysis indicates that puerarin is effective in ameliorating MIRI across various animal models. The findings demonstrate that puerarin administration reduces myocardial infarction size, improves cardiac function, and attenuates key pathological processes, including oxidative stress, inflammation, and cardiomyocyte apoptosis. This indicates that puerarin confers cardioprotection through multi-target mechanisms. Critically, it remains uncertain whether these preclinical effects will translate to clinical settings, and the safety profile of puerarin in humans requires thorough evaluation. Therefore, more rigorous preclinical studies utilizing standardized protocols and well-designed clinical trials are essential to validate these findings and assess the therapeutic potential of puerarin for patients with MIRI.

## Data Availability

The original contributions presented in the study are included in the article/Supplementary Material; further inquiries can be directed to the corresponding author.
