# Supplementary material for: Cardioprotective effects of puerarin against myocardial ischemia–reperfusion injury: a preclinical systematic review and meta-analysis
Source: Front Pharmacol. 2026 Mar 17;17:1770407. doi: 10.3389/fphar.2026.1770407 (PMC13036222; doi:10.3389/fphar.2026.1770407)

Supplementary Material

	Content	Page
**Table S1**	**Representatives search strings for PubMed**	**2**
**Figure S1**	**Funnel plot corrected by the trim-and-fill method**	**3**
**Figure S2**	**Meta-regression plot**	**4**
**Figure S3**	**GRADE Assessment of Evidence Quality for Relevant outcomes**	**5**
**Figure S4-5**	**Publication bias represented by funnel plots**	**6-7**

Table S1.Representatives search strings for PubMed	
**Databases**	**Search strings**
**PubMed**	**((((((((((((((((((((((((((((((((((Myocardial Reperfusion Injury[Title/Abstract]) OR (Myocardial Reperfusion Injuries[Title/Abstract])) OR (Injury, Myocardial Reperfusion[Title/Abstract])) OR (Injuries, Myocardial Reperfusion[Title/Abstract])) OR (Reperfusion Injury, Myocardial[Title/Abstract])) OR (Reperfusion Injuries, Myocardial[Title/Abstract])) OR (Myocardial Ischemic Reperfusion Injury[Title/Abstract])) OR (Myocardial Ischemic Reperfusion Injuries[Title/Abstract])) OR (Myocardial Ischemia Reperfusion Injury[Title/Abstract])) OR (Myocardial Ischemia Reperfusion Injuries[Title/Abstract])) OR (Myocardial Ischemia/Reperfusion Injury[Title/Abstract])) OR (Myocardial Ischemia/Reperfusion Injuries[Title/Abstract])) OR (Myocardial Ischemia-Reperfusion Injury[Title/Abstract])) OR (Myocardial Ischemia-Reperfusion Injuries[Title/Abstract])) OR (MIRI[Title/Abstract])) OR (Myocardial I/R Injury[Title/Abstract])) OR (Myocardial I/R Injuries[Title/Abstract])) OR (Myocardial IR Injury[Title/Abstract])) OR (Myocardial IR Injuries[Title/Abstract])) OR (Myocardial Ischemia Reperfusion[Title/Abstract])) OR (Myocardial Ischemia/Reperfusion[Title/Abstract])) OR (Myocardial Ischemia-Reperfusion[Title/Abstract])) OR (Myocardial I/R[Title/Abstract])) OR (Ischemia-Reperfusion Injury, Myocardial[Title/Abstract])) OR (Ischemia-Reperfusion Injuries, Myocardial[Title/Abstract])) OR (Cardiac Ischemia-Reperfusion Injury[Title/Abstract])) OR (Cardiac Ischemia-Reperfusion Injuries[Title/Abstract])) OR (Myocardial Reperfusion Damage[Title/Abstract])) OR (Myocardial Reperfusion Damages[Title/Abstract])) OR (Myocardial Revascularization[Title/Abstract])) OR (Myocardial Revascularisation[Title/Abstract])) OR (Myocardial Reperfusion[Title/Abstract])) OR (Reperfusion Arrhythmias[Title/Abstract])) OR (Myocardial Stunning[Title/Abstract])) AND ((((Puerarin[Title/Abstract]) OR (Pueraria[Title/Abstract])) OR (Pueraria flavonoids[Title/Abstract])) OR (Kakonein[Title/Abstract]))**

Figure S1. Funnel plot corrected by the trim-and-fill method

(A)

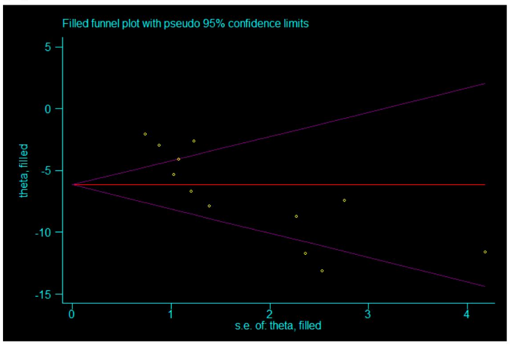

(B)

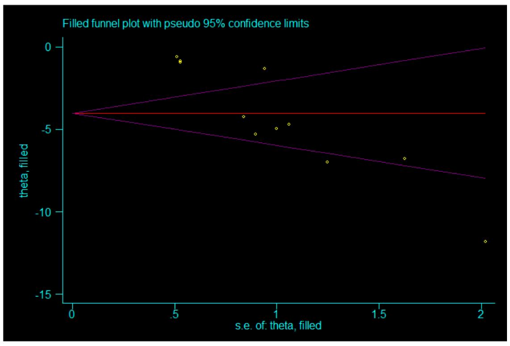

(C)

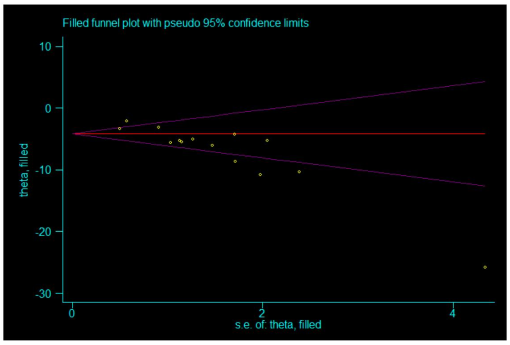

Note:(A)LDH;(B)MDA;(C)Myocardial infarction size

Figure S2. Meta-regression plot

(A)

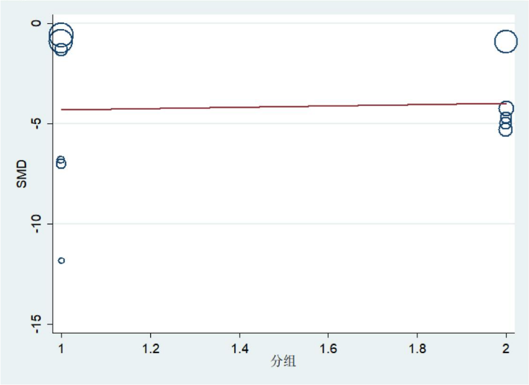

(B)

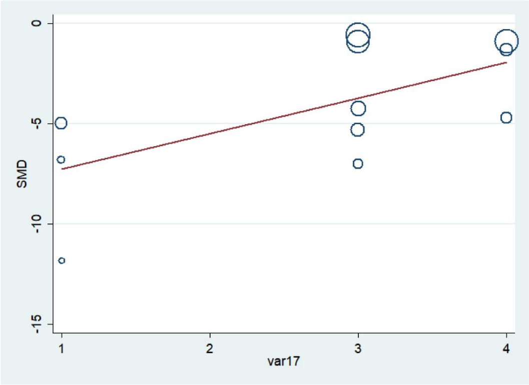

Note:(A)subgroup of dosage;(B)subgroup of route of administration

Figure S3.GRADE Assessment of Evidence Quality for Relevant outcomes

Author(s):
Question: Puerarin compared to PLACEBO OR NONE for Myocardial Ischemia-Reperfusion Injury
Setting:
Bibliography:

Certainty assessment							№ of patients		Effect		Certainty	Importance
№ of studies	Study design	Risk of bias	Inconsistency	Indirectness	Imprecision	Other considerations	Puerarin	PLACEBO OR NONE	Relative (95% CI)	Absolute (95% CI)		
**LDH**												
12	randomised trials	serious	not serious	not serious	not serious	none	77	77	-	SMD **5.55 lower** (7.24 lower to 3.87 lower)	⊕⊕⊕◯ Moderate	CRITICAL
**MDA**												
11	randomised trials	serious	not serious	not serious	not serious	none	90	93	-	SMD **3.77 lower** (5.26 lower to 2.28 lower)	⊕⊕⊕◯ Moderate	CRITICAL
**Myocardial infarction size**												
14	randomised trials	serious	not serious	not serious	not serious	none	112	114	-	SMD **5.41 lower** (6.8 lower to 4.02 lower)	⊕⊕⊕◯ Moderate	CRITICAL
**AI**												
10	randomised trials	not serious	not serious	not serious	not serious	none	94	94	-	SMD **2.76 lower** (3.48 lower to 2.03 lower)	⊕⊕⊕⊕ High	CRITICAL
**CK-MB**												
10	randomised trials	not serious	not serious	not serious	not serious	none	60	60	-	SMD **4.35 lower** (5.59 lower to 3.11 lower)	⊕⊕⊕⊕ High	CRITICAL
**GSH**												
2	randomised trials	serious	not serious	not serious	serious	none	18	18	-	SMD **2.72 higher** (0.57 lower to 6.01 higher)	⊕⊕◯◯ Low	IMPORTANT
**LVDP**												
2	randomised trials	serious	serious	not serious	not serious	none	16	18	-	SMD **4.65 lower** (16.52 lower to 7.22 higher)	⊕⊕◯◯ Low	IMPORTANT
**NO**												
5	randomised trials	not serious	serious	not serious	serious	none	44	44	-	SMD **4.15 higher** (1.93 higher to 6.37 higher)	⊕⊕◯◯ Low	IMPORTANT
**Myocardial ischemic size**												
4	randomised trials	not serious	not serious	not serious	not serious	none	33	33	-	SMD **0.8 lower** (1.31 lower to 0.28 lower)	⊕⊕⊕⊕ High	CRITICAL
Certainty assessment							№ of patients		Effect		Certainty	Importance
№ of studies	Study design	Risk of bias	Inconsistency	Indirectness	Imprecision	Other considerations	Puerarin	PLACEBO OR NONE	Relative (95% CI)	Absolute (95% CI)		
**SOD**												
9	randomised trials	not serious	serious	not serious	not serious	none	79	82	-	SMD **2.48 higher** (1.49 higher to 3.47 higher)	⊕⊕⊕◯ Moderate	CRITICAL
**TNF-α**												
4	randomised trials	serious	not serious	not serious	not serious	publication bias strongly suspected	29	32	-	SMD **3.37 lower** (5.64 lower to 1.1 lower)	⊕⊕◯◯ Low	IMPORTANT

CI: confidence interval; **SMD:** standardised mean difference

Figure S4. Publication bias represented by funnel plots

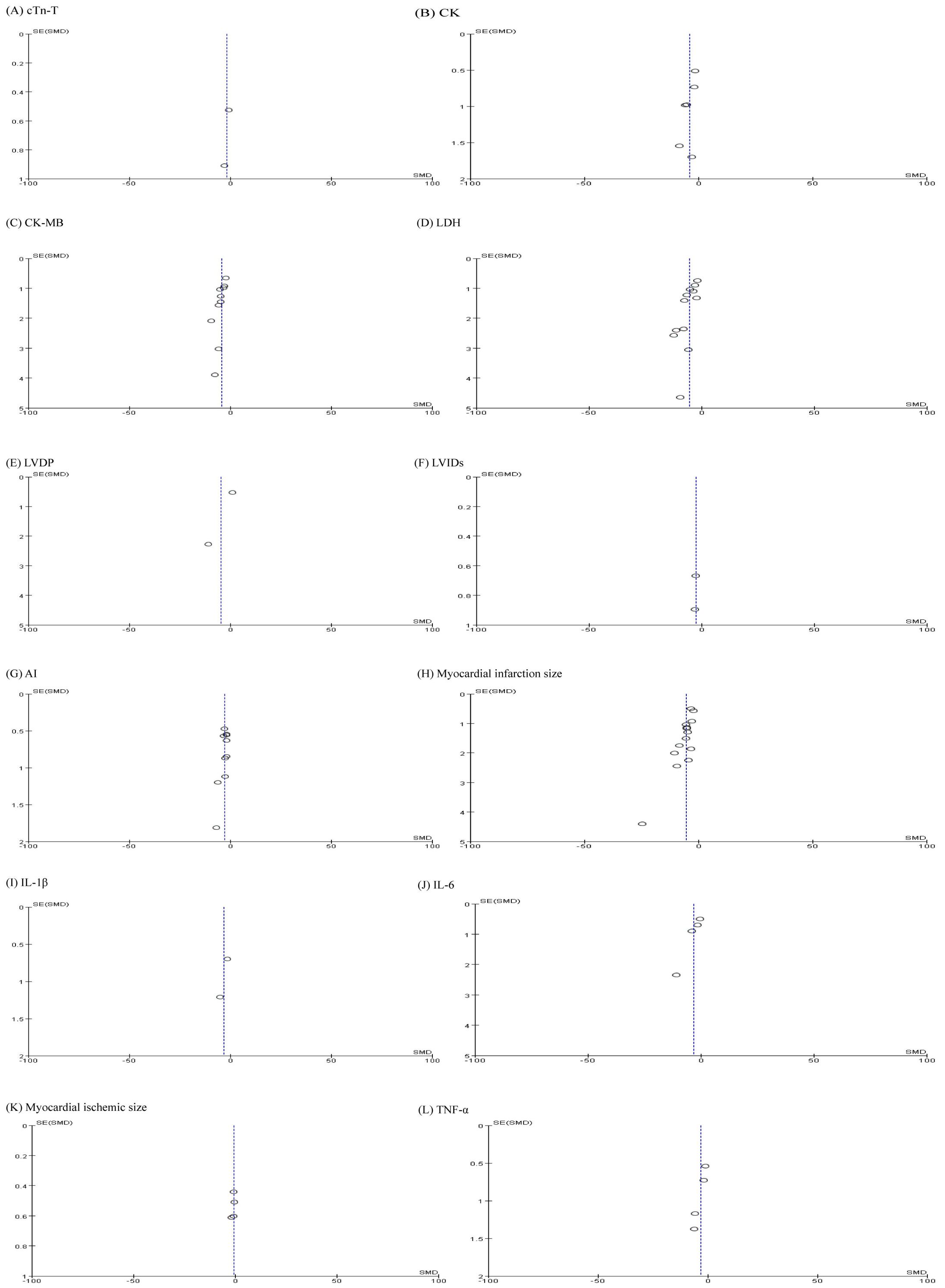

Figure S5. Publication bias represented by funnel plots

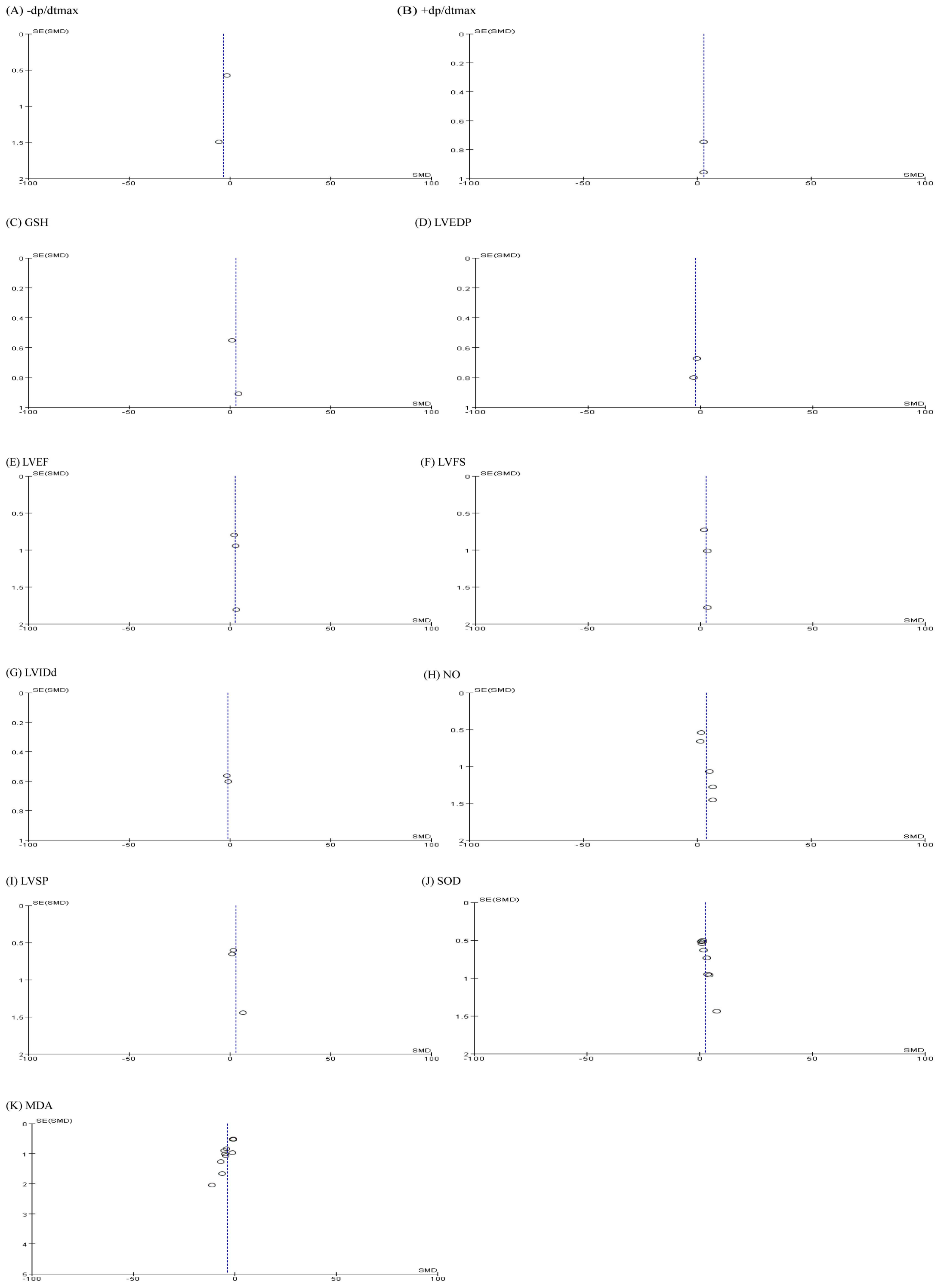

Supplement: Supplementary file 1 [file DataSheet1.pdf]
